# Ultrasound-guided dexmedetomidine combination with modified high fascia iliaca compartment block for arthroscopic knee surgery: what is the optimal dose of dexmedetomidine?

**DOI:** 10.1186/s12871-023-02361-0

**Published:** 2023-12-06

**Authors:** An Chen, Wanqing Duan, Ruijinlin Hao, Chen Wang, Xingguo Xu

**Affiliations:** Department of Anesthesiology, Affiliated Hospital of Nantong University, Nantong, 226001 China

**Keywords:** Modified high fascia iliaca compartment block, Total knee arthroplasty, Postoperative chronic pain

## Abstract

**Background:**

Total knee arthroplasty (TKA) is a common orthopedic procedure for end-stage knee osteoarthritis. Although effective in relieving pain and improving function, postoperative pain is still a common and distressing problem for many patients. This study aims to investigate efficacy of combined administration of dexmedetomidine and modified high fascia iliaca compartment block (H-FICB) in managing acute and chronic pain after TKA, as well as to identify the optimal dosage of dexmedetomidine.

**Methods:**

A double-blind, randomized controlled trial was conducted to evaluate the effects of dexmedetomidine in patients undergoing TKA. A total of 96 patients undergoing TKA were randomly assigned to one of three groups, were treated with different doses of dexmedetomidine All groups received H-FIB. Pain scores, opioid consumption, side effects, and quality of life were recorded 48 h postoperatively.

**Results:**

The intraoperative consumption of remifentanil and propofol in Group D_b_ was significantly reduced compared with that in Group D_0_ and D_a_ (*P* < 0.05). Compared with D_0_ and D_a_ group, D_b_ group had the lowest number of rescue analgesia, analgesia time and morphine accumulative dosage 48 h after operation (*P* < 0.05). The D_b_ group had the lowest scores on the numerical rating scale at rest (*P* < 0.05) and during movement (*P* < 0.01), followed by the D_a_ group and then the D_0_ group. Additionally, the incidence of nausea and vomiting was significantly reduced in the D_b_ group (*P* < 0.05). Furthermore, the D_b_ group had the lowest incidence of chronic pain (*P* < 0.05).

**Discussion:**

In comparison to the other two groups, the administration of combined dexmedetomidine and H-FIB resulted in a significant reduction in pain scores, opioid consumption, and side effects. The optimal dosage of dexmedetomidine was determined to be 1 μg/kg, which provided the most favorable pain relief with minimal adverse effects.

## Introduction

Total knee arthroplasty (TKA) is a widely performed orthopedic surgical procedure used to address various knee joint pathologies [1]. With the increase in the number of TKA surgeries, Anesthesiologists are increasingly focusing on patient satisfaction and comfort after surgery [2]. However, most patients experience varying degrees of pain after surgery, postoperative pain relief had a significant impact on early ambulation, initiation of physical therapy, and improvement in healing [3]. In addition, effective pain management can reduce the risk of hospitalization and thrombosis and improve patient satisfaction. Numerous analgesic techniques, including patient-controlled analgesia (PCA), epidural analgesia, and peripheral nerve blocks, have been demonstrated to be effective in reducing postoperative pain following TKA [4]. Headache and urinary retention are common in patients receiving epidural analgesia [5]. Multimodal analgesia with peripheral nerve blocks has been recommended and considered the best approach for pain management in total knee arthroplasty [6]. However, recent studies have indicated that the fascia iliaca compartment block (FIB) technique might offer superior postoperative pain control following TKA compared to other nerve block techniques such as femoral nerve block (FNB) or adductor canal block (ACB) [7]. Now, the analgesic effects of modified high fascia iliaca compartment block were more significant and had fewer side effects than traditional iliac fascia nerve block [8]. In addition, the use of adjunctive medications such as dexmedetomidine and dexamethasone has been found to enhance the analgesic effect of peripheral nerve blocks and reduce opioid consumption [9–11]. Among these adjunctive medications, the use of intravenous [12] or local infiltration of the α_2_-adrenergic agonist [13], dexmedetomidine has gained increasing attention due to its potential to enhance the quality and duration of analgesia [12, 14]. However, the optimal dose of dexmedetomidine for use in combination with the modified high fascia iliaca compartment block (H-FICB) for postoperative pain management after TKA remains unclear.

Chronic postoperative pain (CPSP) was defined as pain that occurred after surgery and persisted for at least 2 months. In the context of TKA, chronic pain refers to persistent pain that lasts for more than three months after the surgical procedure and proves challenging to alleviate [15]. Research has found that approximately 10–34% of patients experience long-term unpleasant pain after TKA [16], including hyperalgesia, burning, and tingling sensations [17]. These symptoms may be caused by surgical trauma, postoperative inflammatory reactions, nerve compression, or other factors [18].

Therefore, the purpose of this study is to investigate the optimal dose of dexmedetomidine when combined with H-FICB for postoperative pain management after TKA. Specifically, we aim to compare the efficacy and safety of different doses of dexmedetomidine (e.g., 0.25 μg/kg, 0.5 μg/kg, and 1 μg/kg) when used in combination with H-FICB for postoperative pain management after TKA. This study has the potential to provide valuable insights into the optimal use of dexmedetomidine as an adjunct to H-FICB for postoperative pain management after TKA.

## Methods

### Patients

This prospective study included a total of 120 patients who were scheduled for elective TKA and were enrolled at the Affiliated Hospital of Nantong University from October 2022 to December 2022. Study approval was obtained from the Institutional Review Board of Ethics Committee of Affiliated Hospital of Nantong University (number: 2022-K023) and the PODCAST trial is registered with clinicaltrials.gov, number NCT05533970 on 09/09/2022. Patients and their family members were informed about the treatment and signed a consent form. The trial was conducted in accordance with the Declaration of Helsinki.

The inclusion criteria for participants in this study were as follows: patients aged between 18 and 65 years, patients with an American Society of Anesthesiology (ASA) score of I, II, or III, and a body mass index (BMI) ranging from 18 to 38 kg/m2. The primary exclusion criteria included the presence of cerebrovascular diseases, coagulopathy, complications related to mental illness, hepatic, renal, or cardiorespiratory failure, allergy to local anesthetics, and pregnancy.

### Randomization and blinding

Randomization tables generated by computers randomly assigned patients to three groups (n = 32) to receive 30 mL of plain ropivacaine 0.375% plus 0.25 μg/kg dexmedetomidine (D_0_ group), 30 mL of plain ropivacaine 0.375% plus 0.5 μg/kg dexmedetomidine (D_a_ group), or 30 mL of plain ropivacaine 0.375% plus 1 μg/kg dexmedetomidine (D_b_ group). The investigator who creates the random sequence is the investigator who logs in and assigns study patients to study groups according to the random list.

Group work was hidden in sealed, numbered, opaque envelopes until the day of surgery. The anesthesiologist’s assistant opened each bag and prepared appropriate study medication. This anesthesiologist is no longer involved in research or patient care. Anesthetists who performed postoperative patient assessments, surgeons, physical therapists, acute pain nurses, and researchers were blinded to treatment group assignment.

### Anesthesia

All patients were intubated through the peripheral vein of the arm in the anesthesia preparation room, and blood oxygen saturation (SpO_2_), heart rate (HR), end-tidal carbon dioxide (EtCO_2_), invasive blood pressure (IBP) and electrocardiogram (ECG) were routinely monitored. The modified high fascia iliaca compartment block was administered 30 minutes prior to surgery. The patient was positioned supine, and the anterior superior iliac spine was identified as a reference point, 5 cm below which the iliac crest was determined. Using ultrasound guidance, the ‘bow-tie sign’ was identified, and the puncture site was disinfected with a skin disinfectant. Various drug concentrations were utilized based on the assigned groups. After entering the operating room, anesthesia was induced with midazolam 0.15 mg/kg, propofol 4 mg/kg, sufentanil 0.25 μg/kg, cisatracurium 0.2 mg/kg Intubation. While propofol and remifentanil were used for the maintenance of anesthesia. Intraoperatively, Lactated Ringer’s solution was administered at a rate of 6 to 8 ml/kg per hour. The consumption of remifentanil and propofol during operation was recorded.

### Assessment of outcomes

Degree of pain was evaluated by the numerical rating scale at rest (NRS.R) and during movement (NRS.M) at the same time points. The patients were provided with patient-controlled intravenous anesthesia (PCIA) devices contained 100-mg morphine (1 mg/ml) without continuous infusion and set with a lock-out time of 15 min. Patients were instructed to use the device by pushing the button when needed. The devices were set to a maximum dose of 20 mg morphine per day. The post-operative button-push count for PCIA demand and PCIA usage dosage were calculated and recorded at the first 48 h postoperative.

We systematically assess and record the occurrence of adverse events every 8 h was conducted to determine the incidence of adverse events following surgery, including nausea, vomiting, hypotension, bradycardia, arrhythmia, respiratory depression, mechanical ventilation, pruritus, and sedation. Using the three-point scale (1 = mild, 2 = moderate, 3 = severe) above, postoperative nausea, vomiting, and pruritus were assessed.

### Six-month follow-up assessment

During the patients’ visits to the pain clinic in the third and sixth months after surgery, an untrained physician unknowingly assessed them. The assessment included evaluating pain intensity, nature, duration, aggravating and mitigating factors, and analgesic medication. The Leeds Assessment of Neuropathic Symptoms and Signs (LANSS) Pain Scale was used as the assessment tool to evaluate chronic neuropathic pain in this study [19].

### Sample size calculation

Our primary outcome parameter was the difference in NRS scores at movement during the first 48 h postoperatively. According to the preliminary experiment, expected post-treatment the NRS scores at movement during the first 48 h postoperatively was 3.9 ± 1.2 in group D_0_, 3.5 ± 0.9 in group D_a_ and 2.9 ± 0.5 in group D_b_. We use PASS 15 software to estimate the sample size required for this study, multiple independent samples one-way ANOVA was used. Power analysis indicated that a minimum sample size of 29 patients in each group was needed, with a significance level of 0.05 and a power of 0.8. To account for patient dropout and protocol violations, the final is derived as at least 32 cases per group, we recruited a total of 96 patients.

### Data analysis

The distribution of baseline variables was assessed using the Shapiro-Wilk test. Continuous variables were reported as mean (± SD) and analyzed using one-way ANOVA with post-hoc multiple comparisons. Categorical data were reported as numbers and percentages and analyzed using the chi-squared test or Fisher’s exact test with the Bonferroni correction to calculate adjusted *P*-values. Nonparametric data were analyzed using the Mann-Whitney U test. Statistical significance was defined as a *p*-value less than 0.05. All statistical analyses were performed using IBM SPSS Statistics version 26.

## Result

**Fig. 1:**
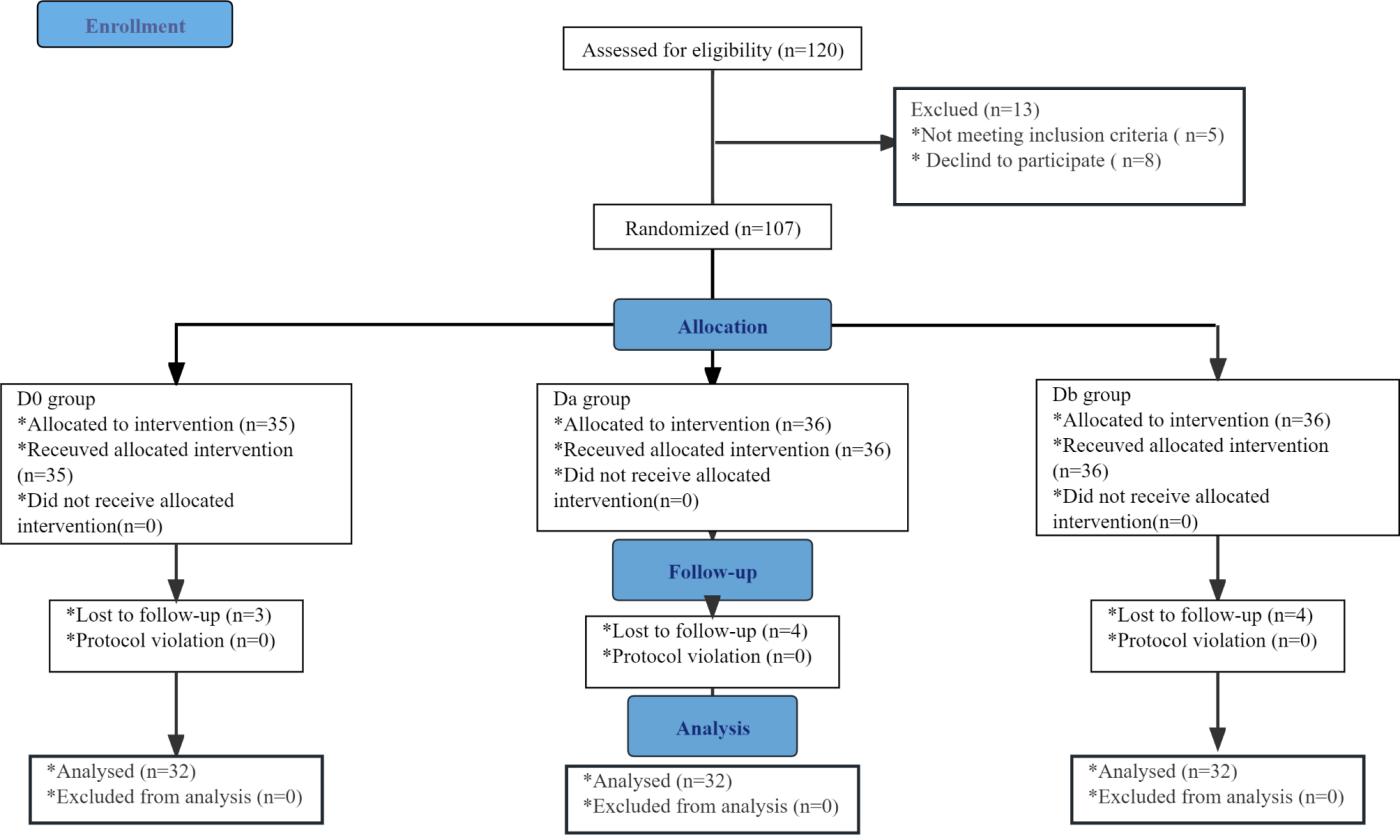
A flow diagram of inclusion and exclusion criteria according to the CONSORT (Consolidated Standards of Reporting Trials) statement

A total of 120 patients were included in the study and each group received the study treatment after randomization. Twenty-four patients were withdrawn from the study due to loss to follow-up, declining to participate, or not receiving the allocated intervention (Fig. 1).

There were no statistically significant differences in age, sex, side of operation, body mass index (BMI), operative time, or between the three groups (Table 1). Compared withD_0_, D_a_ Group, the intraoperative consumption of remifentanil and propofol in Group D_b_ was significantly decreased (*P* < 0.05) (Table 1).

**Table 1:** Patients’ Demographic and Clinical Data

Item	D_0_ Group (n = 32)	D_a_ Group (n = 32)	D_b_ Group (n = 32)	*P*
Age, y	69.03 ± 6.87	69.60 ± 5.37	67.25 ± 5.83	*P* = 0.242
Sex, no. male/no. female	17/15	16/16	15/17	*P* = 0.623
BMI, kg/m^2^	22.81 ± 1.62	22.94 ± 1.50	22.44 ± 1.74	*P* = 0.357
ASA I/II/III	1/26/5	0/26/6	1/28/3	*P* = 0.525
Side of operation (right/left)	14/18	16/16	15/17	*P* = 0.591
Duration of surgery, min	91.44 ± 8.29	87.66 ± 9.68	84.81 ± 7.80	*P* = 0.121
Total remifentanil(mg)	1.18 ± 0.10	1.15 ± 0.05	1.13 ± 0.06	*P* = 0.013
Total propofol (mg)	210.59 ± 15.18	196.66 ± 13.84	187.50 ± 8.37	*P* = 0.001*

**Note**: The data are expressed as the mean ± SD. ^*^P < 0.05 versus D_0_ group

Abbreviations: BMI, body mass index; ASA, American Society of Anesthesiologists

### Usage condition of PCIA in the first 48 h postoperative

In the D_b_ group, fourteen patients (43.8%) required rescue analgesics within the first 48 h after surgery. In the D_0_ group, twenty-five patients (78.1%) required postoperative rescue analgesia, and in the D_a_ group, twenty patients (62.5%) required it (*P* < 0.001). The cumulative analgesia time and morphine consumption were measured as 8.03 ± 3.66 h and 10.24 ± 4.34 h and 13.12 ± 2.51 mg and 8.60 ± 2.85 mg in the D_0_ and D_a_ groups, respectively, compared to 19.95 ± 2.78 h and 4.14 ± 1.01 mg in the D_b_ group (*P* < 0.001) (Table 2).

**Table 2:** Consumption of Rescue Analgesic Medications in the First 48 h Postoperative

Item	D_0_ Group (n = 32)	D_a_ Group (n = 32)	D_b_ Group (n = 32)	*P*
Analgesia time, h	8.03 ± 3.66(n = 25)	10.24 ± 4.34(n = 20)	19.95 ± 2.78(n = 14)	P_a_=0.057
The postoperative button-push count for PCIA times				P_b_<0.001*
				P_c_<0.001*
No request	7(21.9%)	12(37.5%)	18(56.2%)	
1–5	0(0%)	3(9.4%)	12(37.5%)	
6–10	5(15.6%)	11(34.4%)	2(6.3%)	P_a_<0.001*
> 10	20(62.5%)	6(18.8%)	0(0%)	P_b_<0.001*
Cumulative morphine consumption in 1st 48 h postoperative, mg	13.12 ± 2.51	8.60 ± 2.85	4.14 ± 1.01	P_c_<0.001*

**NOTE**: Data are express as mean ± SD and number. ^*^P < 0.001; P_a_, significance between D_0_ and D_a_ groups; P_b_, significance between D_0_ and D_b_ groups; Pc, significance between D_a_ and D_b_ groups

**Fig. 2:**
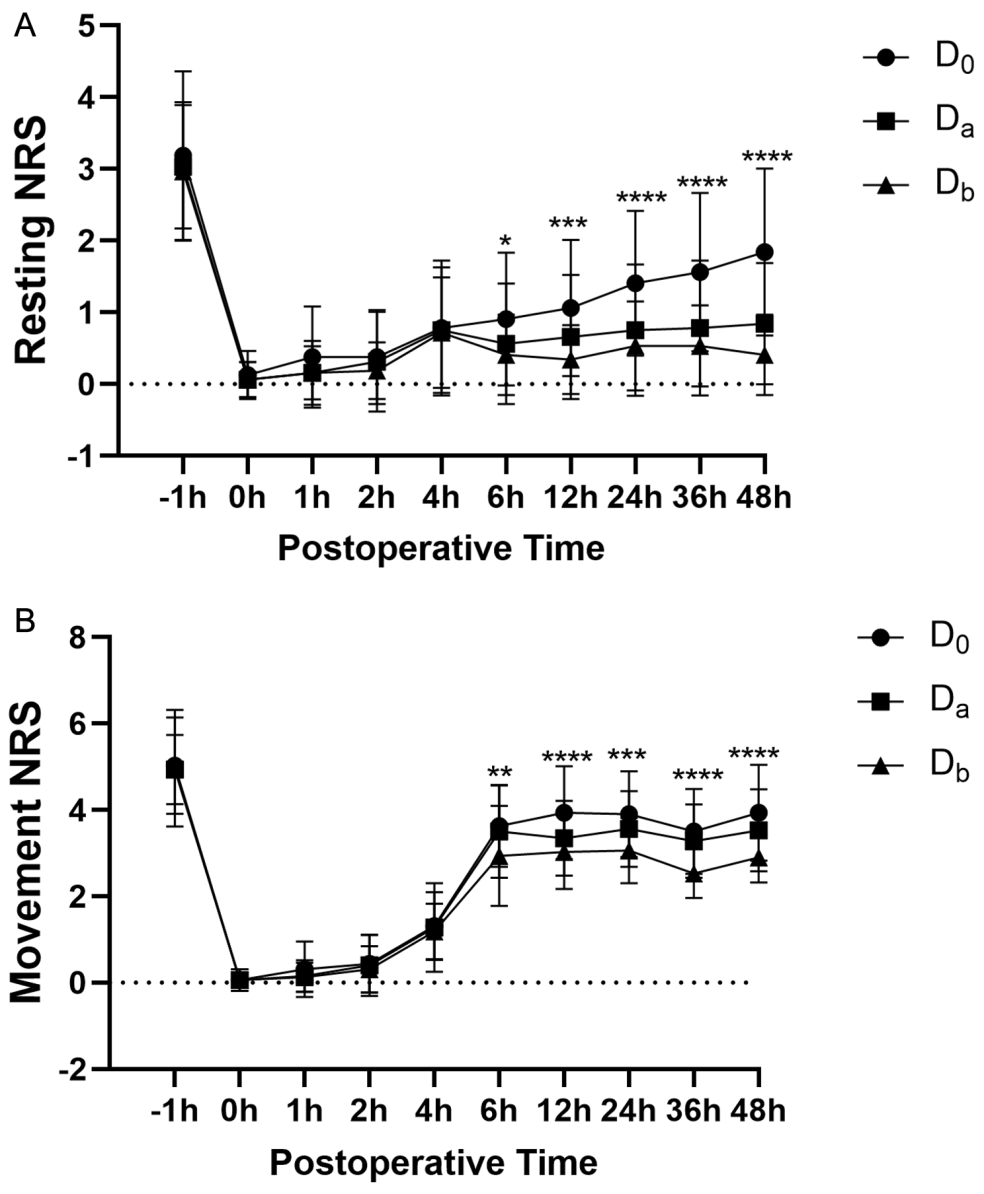
(**A**) NRS.R scores in the three groups at different postoperative periods. A line graph represents the results. ^*^P < 0.05, ^**^P < 0.01, ^***^P < 0.001, ^****^P < 0.0001 compared with Group D_0_. (**B**) NRS.M scores in the three groups at different postoperative periods. A line graph represents the results. ^*^P < 0.05, ^**^P < 0.01,^***^P < 0.001,^****^P < 0.0001 compared with Group D_0_ Abbreviation: NRS.R, Numeric Rating Scale of Rest; NRS, Numeric Rating Scale of Movement

There were no significant differences observed between the groups in terms of the mean NRS.R scores at recovery (*P* = 0.494) and the postoperative 4th hour (*P* = 0.819). However, starting from the 6th hour postoperatively and continuing until the 48th hour, the D_b_ group consistently demonstrated the lowest NRS.R scores, followed by the D_a_ group, and finally the D_0_ group (*P* < 0.05). Similarly, during the same time period (6th to 48th hour), the D_b_ group exhibited the lowest mean NRS.M scores, followed by the D_a_ group, and then the D_0_ group (*P* < 0.01) (Fig. 2, A and B).

Compared to the other two groups, D_b_ group, the number of cases of nausea and vomiting after surgery was significantly reduced (*P* < 0.05), and there were no symptoms of delay in awakening, or respiratory depression. (Table 3) No instances of severe vomiting, nausea, or pruritus were reported in any of the study groups.

**Table 3:** Adverse events in patients

Item	D_0_ Group (n = 32)	D_a_ Group (n = 32)	D_b_ Group (n = 32)	*P*
Nausea	11(34.4%)	5(15.6%)	1(3.1%)	*P* = 0.001*
Vomiting	8(25%)	4(12.5%)	2(6.1%)	*P* = 0.003*
Pruritus	6(18.8%)	4 (12.5%)	1(3.1%)	*P* = 0.006*
Delay in awakening	0(0%)	0(0%)	0(0%)	*P* = NA
Respiratory depression	0(0%)	0(0%)	0(0%)	*P* = NA

**NOTE**: ^*^P < 0.05 versus D_0_ group

### Chronic pain assessments

The mean LANSS score was found to be the lowest in the D_b_ group during the third postoperative month (5.44 ± 2.03 vs. 7.72 ± 3.41 and 9.72 ± 2.96, *P* < 0.05), as well as the sixth postoperative month (5.09 ± 2.41 vs. 7.53 ± 3.08 and 9.66 ± 3.16, *P* < 0.05). In comparison to the D_0_ and D_a_ groups, the D_b_ group demonstrated a significant reduction in the occurrence of neuropathic pain (LANSS score ≥ 12) during the third and sixth postoperative months. Only one patient in the D_b_ group reported neuropathic pain, while nine and four patients in the D_0_ and Da groups, respectively, reported such pain. No significant differences were observed between the D_0_ and D_a_ groups (Table 4).

**Table 4:** LANSS Score in the Third and Sixth Months Postoperatively

	D_0_ Group (n = 32)	D_a_ Group (n = 32)	D_b_ Group (n = 32)	
LANSS (3)				P_a_=0.004
Total score (mean ± SD)	9.72 ± 2.96	7.72 ± 3.41	5.44 ± 2.03	P_b_<0.001
<12	23	28	31	P_c_=0.006
≥ 12	9	4	1	
LANSS (6)				
Total score (mean ± SD)	9.66 ± 3.16	7.53 ± 3.08	5.09 ± 2.41	P_a_=0.004
<12	23	28	31	P_b_<0.001
≥12	9	4	1	P_c_=0.001

**NOTE**: Data are express as mean ± SD and number. LANSS (3), LANSS score in the third month postoperative; LANSS (6), LANSS score in the sixth month postoperative; P_a_, significance between D_0_ and D_a_ groups; P_b_, significance between D_0_ and D_b_ groups; P_c_, significance between D_a_ and D_b_ groups

## Discussion

The objective of this study was to assess the impact of three different doses of topical dexmedetomidine (0.25 μg/kg, 0.5 μg/kg, and 1 μg/kg) in conjunction with H-FICB on acute pain management and the incidence of chronic pain in patients undergoing total knee arthroplasty. The study findings indicated that all three treatment groups demonstrated efficacy and tolerability, with few systemic serious adverse effects. Notably, patients administered with a dosage of 1 μg/kg dexmedetomidine displayed the lowest average postoperative NRS scores and the lowest average LANSS scores throughout the first to sixth postoperative months.

Our results showed that this combination technique provided effective postoperative pain control, with lower pain scores and reduced postoperative opioid consumption compared to the control group receiving local anesthetic alone [20]. Moreover, we identified a dose-response relationship between dexmedetomidine and its analgesic effect, with the optimal dose of 1 μg/kg resulting in the greatest pain relief and fewest adverse effects. However, most of the adverse reactions in group D_0_ may be caused by various reasons. The dosage of opioid administration can influence the incidence of post-operative nausea and vomiting (PONV). Due to the early occurrence of postoperative pain in group D_0_, more opioids are used. High doses of opioids, especially when administered intravenously, are associated with a greater risk of PONV. Further studies of larger sample size are needed to confirm the dose-dependent effect of opioid on the incidence of PONV.

Dexmedetomidine is a highly selective α_2_-adrenergic receptor agonist [21] that produces potent sedative [10], anxiolytic, and analgesic effects [22]. Its analgesic mechanisms involve decreasing the release of norepinephrine and substance P in the spinal cord [13], inhibiting pain signaling pathways [23], and modulating descending inhibitory pathways. Dexmedetomidine also has anti-inflammatory properties and can attenuate the neuroinflammatory response to surgery [24], which may contribute to its analgesic efficacy in the postoperative period.

Chronic pain after total knee arthroplasty (TKA) refers to persistent pain lasting more than three months post-surgery that poses a challenge for management and relief [15]. The strength of early postoperative pain memory has been proven to be the most important factor in the development of chronic pain [18]. Neural influence may play a significant role in the development of chronic pain following TKA [18], while another study has demonstrated the effectiveness of neural modulators in reducing such pain. These neural modulators include antidepressants [25], antiepileptic drugs [26], and anticonvulsants [27], can alter the transmission of nerve impulses by affecting neurotransmitters, relieving pain, and ameliorating symptoms of neural dysfunction. Rekatsina et al. have demonstrated that perioperative iv infusion of dexmedetomidine had a beneficial effect on the prevention of chronic postoperative pain at 3 months, the exact mechanism of action is still unclear [28]. We think chronic pain conditions are often associated with sympathetic hyperactivity, dexmedetomidine can inhibits the release of norepinephrine, resulting in a reduction of sympathetic nervous system activity [29], also by binding to α-2 receptors in the spinal cord, it reduces the release of excitatory neurotransmitters, such as glutamate, and enhances the activity of inhibitory neurotransmitters, likeγ-aminobutyric acid (GABA) [30]. Chronic pain conditions often involve neuroinflammatory processes, and highest dose of dexmedetomidine’s ability to more suppress pro-inflammatory cytokine release and reduce glial cell activation more help alleviate the chronic inflammatory component of pain [31]. Dexmedetomidine also can produce neuroprotective effects through various mechanisms, such as antioxidation and anti-infection activities, the inhibition of apoptosis, the promotion of neurogenesis, and the influence of cell signaling pathways [32]. Highest dose of dexmedetomidine’s ability to reduce chronic pain is rooted in its multifaceted pharmacological actions. Therefore, further studies of the specific mechanism of action by which the highest doses of dexmedetomidine relieve chronic pain require.

Based on our research, it appears that the H-FICB technique offers a more comprehensive and effective approach to regional anesthesia for knee joint procedures when compared to other methods like femoral nerve block or adductor canal block [3, 20, 33]. This is due in part to its ability to provide a wider range of sensory and motor blockade throughout the surrounding tissues [34]. Furthermore, we have found that by incorporating dexmedetomidine into the local anesthetic mixture used during this procedure, patients can experience even greater pain relief and improved overall outcomes. This combination has been shown to enhance the analgesic effect of the treatment, providing patients with a more comfortable and safe recovery period. Overall, our study suggests that utilizing the H-FICB technique along with dexmedetomidine can offer significant benefits for those undergoing TKA. By providing superior pain management and broader coverage of affected areas, this approach become an important link forward in regional anesthesia techniques for these types of surgeries. In short, the combination of dexmedetomidine and H-FICB has the potential to reduce opioid analgesic requirements, thereby minimizing associated risks such as respiratory depression, sedation, and nausea. Furthermore, this combination may improve postoperative recovery by enhancing patient mobility and reducing the incidence of chronic pain.

## Limitations

Our study has several limitations that should be acknowledged. Firstly, the sample size was relatively small, which may limit the generalizability of our findings. Additionally, the absence of a placebo control group restricts our ability to establish a direct comparison between the intervention and a non-treatment condition. Future investigations incorporating larger sample sizes and including a placebo control group are warranted to validate our results and provide further insights into the long-term effects of this combination therapy. Furthermore, longer follow-up periods and studies exploring the optimal duration and frequency of dexmedetomidine administration would be beneficial in enhancing our understanding of its therapeutic potential. Finally, additional research is warranted to investigate the safety and potential adverse effects of dexmedetomidine, particularly in vulnerable populations such as elderly patients and those with comorbidities.

## Conclusion

In comparison to the other two groups, the administration of combined dexmedetomidine and H-FIB resulted in a significant reduction in pain scores, opioid consumption, and side effects, while also improving patient comfort and enhancing postoperative recovery. The optimal dosage of dexmedetomidine was determined to be 1 μg/kg, which provided the most favorable pain relief with minimal adverse effects. These findings suggest that the combination of dexmedetomidine and H-FICB is a safe and effective approach for pain management following TKA, and a dosage of 1 μg/kg is recommended for optimal analgesic effects.

## Data Availability

The datasets used and/or analyzed during the current study are available from the corresponding author on reasonable request.

## References

[1] Li JF, Li H, Zhao H, Wang J, Liu S, Song Y, Wu HF (2017). Combined use of intravenous and topical versus intravenous tranexamic acid in primary total knee and hip arthroplasty: a meta-analysis of randomised controlled trials. J Orthop Surg Res.

[2] Akesen S, Akesen B, Atici T, Gurbet A, Ermutlu C, Ozyalcin A (2021). Comparison of efficacy between the genicular nerve block and the popliteal artery and the capsule of the posterior knee (IPACK) block for total knee replacement Surgery: a prospective randomized controlled study. Acta Orthop Traumatol Turc.

[3] Allen HW, Liu SS, Ware PD, Nairn CS, Owens BD (1998). Peripheral nerve blocks improve analgesia after total knee replacement Surgery. Anesth Analg.

[4] Fan X, Cao F, Luo A (2021). Femoral nerve block versus fascia iliaca block for pain control in knee and hip arthroplasties: a meta-analysis. Medicine.

[5] Boonmak P, Boonmak S. Epidural blood patching for preventing and treating post-dural puncture headache. Cochrane Database Syst Rev 2010(1):CD001791. 10.1002/14651858.CD001791.pub2 20091522

[6] Karpetas GZ, Spyraki MK, Giakoumakis SI, Fligou FG, Megas PD, Voyagis GS, Panagiotopoulos EC (2021). Multimodal analgesia protocol for pain management after total knee arthroplasty: comparison of three different regional analgesic techniques. J Musculoskelet Neuronal Interact.

[7] Kanadli H, Dogru S, Karaman T, Karaman S, Tapar H, Sahin A, Asci M, Kanadli KA, Suren M (2018). Comparison of the efficacy of femoral nerve block and fascia iliaca compartment block in patients with total knee replacement. Minerva Anestesiol.

[8] Chen L, Shen Y, Liu S, Cao Y, Zhu Z (2021). Ultrasound-guided supra-inguinal fascia Iliaca compartment block for older adults admitted to the emergency department with hip fracture: a randomized controlled, double-blind clinical trial. BMC Geriatr.

[9] Hu X, Li J, Zhou R, Wang Q, Xia F, Halaszynski T, Xu X. Dexmedetomidine added to local anesthetic mixture of Lidocaine and Ropivacaine enhances onset and prolongs duration of a popliteal approach to sciatic nerve blockade. Clin Ther 2017;39(1). 10.1016/j.clinthera.2016.11.011 27955918

[10] Chan IA, Maslany JG, Gorman KJ, O’Brien JM, McKay WP (2016). Dexmedetomidine during total knee arthroplasty performed under spinal anesthesia decreases opioid use: a randomized-controlled trial. Can J Anaesth = J Canadien D’anesthesie.

[11] Moeen SM, Ramadan IK, Elkady HA (2017). Dexamethasone and Dexmedetomidine as an adjuvant to Intraarticular Bupivacaine for Postoperative Pain relief in knee arthroscopic Surgery: a Randomized Trial. Pain Physician.

[12] Jin XB, Xiao R, Zhou W, Liu C, Luo YR, Liu RH, Xu GH, Mei B, Xu JN, Yang R (2021). Effect of Different Modes of Administration of Dexmedetomidine Combined with nerve block on postoperative analgesia in total knee arthroplasty. Pain Ther.

[13] Bhana N, Goa KL, McClellan KJ. Dexmedetomidine. Drugs. 2000;59(2). 10.2165/00003495-200059020-00012 10730549

[14] Lee JJ, Kim DY, Hwang JT, Song DK, Lee HN, Jang JS, Lee SS, Hwang SM, Moon SH, Shim JH (2021). Dexmedetomidine combined with suprascapular nerve block and axillary nerve block has a synergistic effect on relieving postoperative pain after arthroscopic rotator cuff repair. Knee Surg Sports Traumatol Arthrosc.

[15] Wylde V, Bruce J, Beswick A, Elvers K, Gooberman-Hill R (2013). Assessment of chronic postsurgical pain after knee replacement: a systematic review. Arthritis Care Res (Hoboken).

[16] Beswick AD, Wylde V, Gooberman-Hill R, Blom A, Dieppe P (2012). What proportion of patients report long-term pain after total hip or knee replacement for osteoarthritis? A systematic review of prospective studies in unselected patients. BMJ Open.

[17] Jensen TS, Finnerup NB (2014). Allodynia and hyperalgesia in neuropathic pain: clinical manifestations and mechanisms. Lancet Neurol.

[18] Fregoso G, Wang A, Tseng K, Wang J (2019). Transition from Acute to Chronic Pain: evaluating risk for Chronic Postsurgical Pain. Pain Physician.

[19] Bennett M (2001). The LANSS Pain Scale: the Leeds assessment of neuropathic symptoms and signs. Pain.

[20] Wang X, Sun Y, Wang L, Hao X (2017). Femoral nerve block versus fascia iliaca block for pain control in total knee and hip arthroplasty: a meta-analysis from randomized controlled trials. Medicine.

[21] Li J, Wang H, Dong B, Ma J, Wu X (2017). Adding dexmedetomidine to ropivacaine for femoral nerve block inhibits local inflammatory response. Minerva Anestesiol.

[22] Abdallah FW, Brull R (2013). Facilitatory effects of perineural dexmedetomidine on neuraxial and peripheral nerve block: a systematic review and meta-analysis. Br J Anaesth.

[23] Bao N, Tang B. Organ-protective effects and the underlying mechanism of dexmedetomidine. Mediat Inflamm. 2020:6136105. 10.1155/2020/6136105 PMC7232715 32454792

[24] Bao N, Dai D. Dexmedetomidine protects against ischemia and reperfusion-induced kidney injury in rats. Mediat Inflamm, 2020:2120971. 10.1155/2020/2120971 PMC7157761 32317860

[25] Obata H. Analgesic mechanisms of antidepressants for neuropathic pain. Int J Mol Sci 2017;18(11). 10.3390/ijms18112483 PMC5713449 29160850

[26] Pandey CK, Priye S, Singh S, Singh U, Singh RB, Singh PK (2004). Preemptive use of gabapentin significantly decreases postoperative pain and rescue analgesic requirements in laparoscopic cholecystectomy. Can J Anaesth.

[27] Chaparro LE, Smith SA, Moore RA, Wiffen PJ, Gilron I (2013). Pharmacotherapy for the prevention of chronic pain after Surgery in adults. Cochrane Database Syst Rev.

[28] Rekatsina M, Theodosopoulou P, Staikou C (2022). Perioperative Dexmedetomidine or Lidocaine infusion for the Prevention of Chronic Postoperative and Neuropathic Pain after gynecological Surgery: a Randomized, Placebo-Controlled, double-blind study. Pain and Therapy.

[29] Kenney MJ, Larsen BT, McMurphy RM, Mason D, Fels RJ (2014). Dexmedetomidine and regulation of splenic sympathetic nerve discharge. Auton Neurosci.

[30] Lee M (2013). Neurotransmitters and microglial-mediated neuroinflammation. Curr Protein Pept Sci.

[31] Zhao Y, He J, Yu N, Jia C, Wang S. Mechanisms of dexmedetomidine in neuropathic pain. Front NeuroSci 2020;14. 10.3389/fnins.2020.00330 PMC7214625 32431587

[32] Wu J, Vogel T, Gao X, Lin B, Kulwin C, Chen J. Neuroprotective effect of dexmedetomidine in a murine model of traumatic brain injury. Sci Rep 2018;8(1). 10.1038/s41598-018-23003-3 PMC5862953 29563509

[33] Kertkiatkachorn W, Kampitak W, Tanavalee A, Ngarmukos S (2021). Adductor Canal Block Combined with iPACK (Interspace between the popliteal artery and the Capsule of the posterior knee) Block vs Periarticular Injection for Analgesia after total knee arthroplasty: a Randomized Noninferiority Trial. J Arthroplast.

[34] Vermeylen K, Desmet M, Leunen I, Soetens F, Neyrinck A, Carens D, Caerts B, Seynaeve P, Hadzic A, Van de Velde M. Supra-inguinal injection for fascia iliaca compartment block results in more consistent spread towards the lumbar plexus than an infra-inguinal injection: a volunteer study. Region Anesth Pain M. 2019. 10.1136/rapm-2018-100092 30798268

