# Correction: Ultrasound-guided dexmedetomidine combination with modified high fascia iliaca compartment block for arthroscopic knee surgery: what is the optimal dose of dexmedetomidine?

**DOI:** 10.1186/s12871-023-02391-8

**Published:** 2023-12-22

**Authors:** An Chen, Wanqing Duan, Ruijinlin Hao, Chen Wang, Xingguo Xu

**Affiliations:** Department of Anesthesiology, Affiliated Hospital of Nantong University, Nantong, 226001 China

**Correction: BMC Anesthesiol 23, 400 (2023)**

10.1186/s12871-023-02361-0

Following publication of the original article [1], the authors reported an error found in affiliation 1’s organization name. The correct organization name should be “Affiliated Hospital of Nantong University” instead of “The Hospital Affiliated of Nantong University”.

The original article [1] has been updated.
